# Differential impacts of e-portfolio assessment on language learners’ engagement modes and genre-based writing improvement

**DOI:** 10.1186/s40468-022-00156-7

**Published:** 2022-03-15

**Authors:** Natasha Pourdana, Kobra Tavassoli

**Affiliations:** Department of ELT and Translation, Karaj Branch, Islamic Azad University, Karaj Branch, Karaj, 31485-313 Iran

**Keywords:** E-Portfolio assessment, Genre-based writing, Higher-level skills, Learner engagement, Lower-level skills

## Abstract

Portfolio assessment (PA) as an assessment *for* learning (AfL) alternative has been under-represented in second/foreign language acquisition (SLA) research literature. This study examined the potential impacts of electronic PA (e-PA) on English-as-a-Foreign-Language (EFL) learners’ engagement modes in descriptive and narrative genres of writing on Moodle™. To do so, 56 university students were non-randomly selected and assigned into two intermediate-level EFL cohorts. In a pretest-mediation-posttest study, descriptive and narrative writing tasks completed by two groups were subjected to teacher feedback, student reflection logs, and subsequent revision every week. Results of repeated measures ANOVA indicated significant progress in lower-level skills (sentence structure, word choice/grammar, mechanics), and moderate progress in higher-level skills (organization, development) in both groups’ genre-based writing. Results of one-way ANCOVA reported the notable pretest-to-posttest achievement by both groups with no intergroup statistical differences. The content of students’ reflection logs was inductively analyzed for their behavioral, emotional, and cognitive modes of engagement in e-PA. Qualitative data analysis indicated similar writing time intervals and recurrence of revisions as the behavioral mode of both groups. Participants also expressed novelty, low anxiety, and enjoyment as their emotional experiences. In terms of their cognitive experience, the majority agreed upon the applicability of teacher feedback and positive perception of writing improvement in e-PA. Yet, they were critical to regular mismatches between the scopes of teacher assessment and self-assessment, as well as teacher linguistic bias towards certain writing features. Several pedagogical implications of the study promote the facilitating role of e-PA in genre-based academic writing and e-learning contexts.

## Introduction

A writing portfolio is an electronic or print dossier of students’ written scripts which are selected over time and often endorsed with their self-reflective journals. In education, portfolio assessment (hereafter, PA) is often assumed as a better-quality alternative to traditional, product-oriented standardized testing (Benson, 2007; Hirvela and Sweetland, 2005; Kirkpatrick and Gyem, 2012). However, as Condon and Hamp-Lyons (1994) argued, “portfolio has simply been accepted on faith, on writing specialists’ feeling, that the portfolio is simply better” (p. 277).

Several research studies in second/foreign language (L2) have reported the advantages of PA in terms of the L2 teachers’ positive experience with various types of PA (Lam and Lee, 2010; Lee, 2016), the role of PA in boosting L2 learner autonomy, self-regulated learning, social and metacognitive awareness (Aydin, 2010; Behbahani et al. 2011), and the mediation role of PA in revising works-in-progress (Hamp-Lyon and Condon, 2000; Mphahlele, 2022). Despite the reported educational benefits, PA has remained highly controversial in real classroom situations, namely due to L2 teachers’ inflexibility (Xu and Brown, 2016; Willis, 2011), insufficient language assessment literacy (Gan and Lam, 2020), low engagement of students (Lee and Coniam, 2013), its complicated and holistic grading (Song and August, 2002), and the test-driven, dominant culture in most school systems (Lam, 2018a). Therefore, a full practice of PA in L2 settings has constantly faced massive problems which compelled Hyland and Hyland (2019) to call for more in-depth research on its practical aspects which is the concern in the current study.

From the pedagogical perspective, the process-oriented PA approach to L2 writing redefines it as a recursive and metacognitive activity that engages L2 learners in regular thinking about their language progress with a focus on learner reflection (Lam, 2019). As Lam (2018b) speculated, since writing PA sustains students’ close attention to different aspects of language skills development, it nurtures their self-idealization and regulates their active *engagement* (Burner, 2014; Pourdana and Behbahani, 2013). Such L2 learner engagement is even more intensive in electronic PA (hereafter, e-PA) where they are largely served with rich and enjoyable digital resources in the information and communication technologies (ICT). Yet, the evidence in favor of *how* L2 learners engage in e-PA is still anecdotal and under-researched (Barrot, 2021; Hamp-Lyons, 2007; Hamp-Lyons and Condon, 2000). Similarly, SLA studies on how e-PA might enhance English as a Foreign Language (EFL) learner engagement in genre-based academic writing are a few with limited empirical evidence (Hyland, 2007; Lam, 2018a; Pourdana and Asghari, 2021). To void this gap in the literature, therefore, this study aimed to compare the potential impacts of e-PA on EFL university students’ modes of engagement in two academic genres of descriptive and narrative writing and their subsequent writing improvement.

### Literature review

Since the early 1990s, assessment *for* learning (AfL) has reached a widespread audience in educational contexts (Cowie, 2005; Klenowski, 2009; O’Shea, 2020), and likewise, several studies examined how AfL might benefit and self-regulated learning (Brown and Abeywickrama, 2010; Darling-Hammond and McCloskey, 2008; Earl, 2013). As Ramaprasad (1983) presumed, AfL in L2 classrooms not only needs the learners’ perception of *a gap* between a long-term goal and their status quo but also their commitment to bridge the gap to attain their learning goal.

Ideally, either the L2 learners should engage in self-assessment to generate the information about the noticed gap, or the teachers have to explore the gap and provide feedback to the students about it. Ultimately, the action of closing the gap will be taken by fully engaged students in the process of language learning (Sadler, 2010). Yet, in practice, L2 teachers and learners have more critical steps to take. In their AfL practice, L2 teachers have to lift the capacity in the students to diligently discover their learning gaps, engage themselves, and take full responsibility for carrying out remedial actions. Among various alternatives of AfL which has a large potential to generate such self-regulated learning is portfolio assessment.

PA or portfolio-based assessment is grounded in the social constructivism model of learning (Vygotsky, 1987) which proposes that learning happens effectively when L2 learners proactively construct the knowledge of the target language *for* themselves through social interactions, rather than being the passive recipients of the language knowledge. For instance, writing PA reinforces the L2 learners’ “understanding of writing as a socially situated process in academic discourse communities” (Duff, 2010, p. 169). In doing so, it can assess both higher-level writing skills (e.g., textural, discursive) and lower-level writing skills (e.g., writing mechanics, punctuations) in L2 writing progress (Borg, 2003; Ngui et al. 2020; Price et al. 2010; Steen-Utheima and Hopfenbeck, 2018). Moreover, writing PA demands L2 learners to actively engage in bridging the observed gap in their writing performance (Lam, 2019). This is routinely advised through writing reflective journals, diaries, and redrafting (Hamp-Lyons, 2016).

According to Caner (2010) and Chappuis (2014), successful learner engagement depends on *how well* L2 learners understand the goals pronounced in writing PA, *how soon* they picture the distance between their status quo and those goals, and *what* they do to achieve the goals. In the same vein, L2 writing teachers are recommended to prime self-reflectiveness through ‘scaffolding’ the students in terms of tutorials to the entire PA process (Carless and Boud, 2018; Kusuma et al. 2021), using examples and prompts (Gregory et al., 2001), extending deadlines to engage students even more (Lam, 2014), and informing them of the assessment rubrics (Panadero and Romero, 2014).

L2 learner engagement in PA is not an option but a survival kit. According to Fredricks and Eccles (2002), learner engagement unites three major components of behavior, emotion, and cognition. *Behavioral* engagement implies the learners’ active participation in terms of involvement in on-task behavior and social activities which likely cause positive academic outcomes. *Emotional* engagement entails the learners’ both positive and negative reactions to the teacher feedback, classroom tasks, classmates, academics, and school which influence their willingness to communicate and learn. Finally, *cognitive* engagement guarantees learners’ cognitive investment which mediates their thoughtfulness and openness to self-regulated learning and mastery of challenging skills (Fredricks et al. 2004). Implied in the concept of learner engagement, the integration of behavior, emotion, and cognition seems invaluable to L2 learning as it causes an extended commitment in students (Ecclestone, 2007; Hargreaves, 2005).

From the educational technology perspective, with the rapid surge of ICT in language teaching and assessment, writing portfolios have been transformed from printed folders to online environments to easily archive, distribute, and assess the students’ written works (Barrett, 2007). Similarly, the e-PA incorporates various electronic technologies to generate a stimulating environment by helping the students to collect, organize, and revise their writing e-PA in a variety of formats (Baturay and Daloğlu, 2010). Moreover, the e-PA serves L2 learners with multimedia modalities to display their artifacts with sound, images, and videos, to engage in dynamic self-assessment, and to set metacognitive goals (Hung, 2012). In other words, e-PA has the potential to expand various aspects of students’ e-literacy, including their performance on genre-based academic writing.

Genre-based writing such as narrative, descriptive, or expository is one of the critical issues in students’ academic achievement (De Fina and Georgakopoulou, 2015; Ismailov and Laurier, 2021; Yu, 2020). Derewianka (2003) formulated the writing genres as “the conventional and recurring patterns of everyday, academic and literary texts within a particular culture” (p. 133), and Hyland (2018) defined a genre as “a schema of prior knowledge [or a set of conventions] which we share with others and can draw on to express ourselves efficiently and effectively” (p. 2360). While the process-oriented approach to academic writing has an eye on the L2 writer’s overflow of ideas, the genre-based approach has switched its focus to the socio-literacy of the L2 writers in generating *real* texts that properly address the target discourse community (Hyland, 2007; Saadatmandi et al. 2018). In other words, genre-based writing shares its root in Vygotskyan social constructivism with writing PA. In line with a genre-based approach to PA, EFL learners may have a chance to engage in gaining control over a variety of genre-based writing skills in the target language discourse. However, the research literature of PA mostly pertained to assessing the students’ general writing performance in L1 (Hamp-Lyons, 2016) or L2 (Gottlieb, 2000), with marginal focus on the L2 learners’ genre-based writing performance and their strengths and weaknesses to reach the intended goals. Therefore, an urge for further research on this topic inspired the current study.

In this study, we investigated the impact of e-PA on higher and lower-level writing skills in descriptive and narrative genres of writing by an in-depth analysis of the weekly writing performance of 56 EFL undergraduate students, and their writing achievement through the pretest-posttest summative assessment. Moreover, the impact of e-PA was explored on the participants’ behavioral, emotional, and cognitive modes of engagement, following Fredricks and Eccles’s (2002) learner engagement model. To this end, the following research questions were raised:

- Does writing e-PA have differential impacts on EFL learners’ control of higher and lower-level skills in their descriptive and narrative writing?
- Does writing e-PA have differential impacts on EFL learners’ descriptive and narrative writing achievement?
- How does writing e-PA affect EFL learners’ engagement at behavioral, emotional, and cognitive modes?

## Method

### Context and participants

This study was an extra-curricular course of genre-based writing conducted virtually at a large university campus in the mid-COVID-19 pandemic in Iran. A sample of 56 Persian-speaking EFL freshman students of non-English majors voluntarily took part in this study. A convenience sampling method was adopted (Ames et al. 2019) to select a large enough sample of informants with adequate experience of genre-based writing in English. The selected participants had already performed on at least 10 descriptive and narrative writing tasks as partial requirements in previous English writing courses at university.

The participants’ English proficiency level was measured by administering Oxford Placement Test (Version 1, 2001). Due to the restrictions imposed by the COVID-19 lockdown, we converted its 60 items into Google Forms™, a free web-based survey administration software, for virtual participation. After signing the consent form, the volunteers (*N* = 71) worked on the electronic version of OPT in 45 min and those candidates whose OPT scores determined their language proficiency at intermediate level (30-37, B1 in OPT ranking system, *M* = 35.78, SD = .73, Cronbach’s α = .802, representing strong test reliability) were selected. Next, the participants were randomly split into a group who performed narrative writing tasks (hereafter, NWG) (*N* = 28), and a group who performed descriptive writing tasks (hereafter, DWG) (*N* = 28). After group assignment, we compared the OPT scores of the two groups and found no significant intergroup differences, *t* (55) = .395, *p* = .77.

Other requirements for participating in this study were having a smartphone, accessing the Internet, and being enrolled in the Moodle™ e-course. Our rationale behind selecting Moodle was its user-friendliness. As an open-source learning management system (LMS), Moodle requires no high-tech skills and allows its users to upload and archive their artifacts in multiple formats. Therefore, it seemed a suitable e-PA platform.

The researchers in this study were university professors majoring in teaching English as a foreign language (TEFL) with 15 years of professional experience. Additionally, an MA graduate of English language teaching (ELT) collaborated with the researchers in providing genre-based feedback to the descriptive and narrative writing task outputs, rating the pretest and posttest writings, and analyzing the content of the reflection logs. She had 7 years of experience in teaching general English at private language schools. Table 1 summarizes the attributes of the EFL learner participants.

**Table 1:** Demographic attributes of the participants (%)

Participant	Gender	f	Age range	f	Major	f	Studying English	f	OPT score range	f
*N* = 56	Female, (53.57)	30	20–24, (64.28)	36	Management, (41.07)	23	13 years, (46.42)	26	30–33, (35.71)	20
	Male, (46.42)	26	25–31, (35.71)	20	Economy, (37.30)	21	12 years, (53.57)	30	34–37, (64.28)	36
					Mathematics, (10.81)	6				
					Accounting, (10.81)	6				

### Materials and instruments

#### West Virginia Department of Education (WVDE) writing rubric

The West Virginia Department of Education (WVDE) writing rubric (2011) was adopted as the rating reference for both genre-based teacher feedback and students’ self-assessment of their narrative and descriptive writing performance (Appendix A). The WVDE rubric addresses two higher-level components of *organization* and *development*, and three lower-level components of *sentence structure, word choice*, and *grammar*, and *mechanics*, on a 6-band score, ranging from 1 (minimal) to 6 (exemplary) writing quality criteria. The WVDE writing rubric is commonly known for meeting the criteria in assessing academic writing in the EFL context, as their reasonable cut-off scoring system ensures a reliable impression of student writing performance in English (NBCT Office of Assessment West Virginia Department of Education, 2015). Our logic behind adopting this rubric was its user-friendliness, clarity of rubric indicators, and creditability. The writing rubric was distributed to the participants after briefing of its components.

#### Genre-based elicitation and assessment tasks

The academic goal for choosing the descriptive genre of writing was to enhance the students’ ability in describing tables, figures, flowcharts, and other course-related descriptive writing tasks at the university level. The academic goal for choosing the narrative genre of writing was likewise to develop the students’ writing ability in reporting the stepwise experimental procedures in their assigned projects.

The narrative and descriptive elicitation and assessment tasks were developed after a topic familiarity checklist was distributed among the participants to determine their common grounds. The selected topics had a wide range, including marriage, education, jobs, national holidays, and personal hobbies. The elicitation and assessment tasks were prompted with multimedia input such as diagrams, pictures, and videos to engage the participants as much as possible.

The DWG and NWG participants completed the descriptive writing pretest and posttest, and the narrative writing pretest and posttest tasks, respectively. The assessment tasks input was a short video about the national holiday of Nowruz. The participants in DWG described the popular Nowruz ceremonies in their local community, and the NWG narrated their best experience of Nowruz in 30 min.

#### Reflection logs

Student reflection logs were used as the resource of qualitative data in this study. After the participants received the teacher feedback on every written script, they prepared their reflections logs. They were required to respond to a set of prompts in their reflection logs which addressed the three modes of learning engagement in e-PA.

Informed by Fredricks and Eccles’s (2002) model of learner engagement, the behavioral mode of engagement was operationally defined as the recurrence of revisions and the length of the time every participant spent on an assignment. The participants were required to download their assignment embedded with teacher feedback from their Moodle profile and revise it accordingly as often as they wished. They were also required to re-post their draft every time they revised it and report the exact amount of time they spent on preparing the revisions.

The first reflection log prompt which addressed the behavioral mode of engagement was *How much time did you spend on writing and revising your drafts on Task #?* The second prompt addressed the emotional mode of engagement and required the participants to retrospect on their positive and negative personal experiences they had after every task they completed and every teacher feedback they received, by replying to *How do you describe your experience of working on Task #?* The third prompt required the participants to report on their learning experience, writing weaknesses, quality of teacher feedback on every task they completed by responding to *What did you learn from the teacher feedback and how did you apply it to Task #?*

The students were allowed (but not recommended) to write their logs in Persian (their L1) to express their thoughts easily and clearly. As a result, in the submitted reflection logs, a large body of Persian words (*N* = 560) was written which were encoded similarly to the English reflection logs. The encoding and interpretation of reflection logs were done collaboratively by the researchers and reached a 93% agreement. The controversies were negotiated case by case to the full consensus.

### The procedure of data collection and analysis

The blueprint of writing PA is operationalized in four stages: collection, selection, reflection, and teacher delayed evaluation of students’ writings (Lam, 2019). In a typical PA system, the *collection* is the gradual compilation of students’ multiple written drafts. *Selection* is the students’ self-collection of the best pieces of their work for the teacher’s final grading. *Reflection* is the student self-assessment of their own personal and learning experience, and *teacher delayed evaluation* is assigning grades on the final written drafts by the teacher. Yet, SLA researchers are allowed to modify this framework to make it compatible with the purpose of their study or to cope with other limiting contextual factors (Hamp-Lyons and Condon, 2000). In this study, therefore, we deliberately omitted the selection step to collect as much data as we could.

The study commenced with a two-hour webinar on the purpose and focus of e-PA, frameworks of descriptive and narrative writing tasks by presenting two anchor essays, the WVDE writing rubrics, and the process of writing reflection logs by responding to the three prompts for every assignment task and posting them on the personal Moodle profiles. After administering the OPT and the group assignment, the NWG and DWG participants were pretested with narrative and descriptive writing tasks, respectively. During the 6-week online writing course, they completed the assignment tasks every week and posted their writing to their Moodle profile. Within 24 h, we provided them with corrective feedback directly pointed to their committed errors, and some suggested comments on how to revise them. The revised drafts had to be posted as soon as possible, but the students could re-do the revisions until they were satisfied with their task outcome. Also, they archived the recurrent revisions as new document files to let us know the frequency of revisions. On every assignment task, the students wrote a reflection log by responding in detail to the three prompts, either in L2 or L1. The course was ended with the final performance of NWG and DWG on the 30-minute narrative and descriptive posttest tasks, respectively.

On every assignment task outcome, the researchers accumulated the feedback points regarding the higher-level (i.e., organization, and development) and lower-level (i.e., sentence structure, word choice and grammar, and mechanics) writing skills inhered in the WVDE writing rubric, and announced them on the individual participant’s private Moodle profile.

The narrative and descriptive pretest and posttests were likewise rated based on the WVDE 6-band scale. Therefore, every participant received six feedback scores on the completed assignment tasks and two gain scores on the pretest and posttest. The collected e-portfolios were evaluated holistically by assigning them a letter A, B, or C, based on the overall quality of the revised final drafts and completeness of the submitted portfolios and reflection logs.

## Results

The pool of quantitative data (collected feedback points) was keyed into Statistical Package for Social Sciences (SPSS) version 25. The significance level in statistical analysis was set at 0.05. To address the first research question, the researchers initially conducted descriptive statistical analysis and tests of normality followed by repeated-measures analysis of variance (RM ANOVA) to identify potential differences in the feedback points on the descriptive and narrative writing tasks completed by DWG and NWG over 6 weeks. It should be noted that the observed *decrease* in feedback points was interpreted as the students’ progress in their writing ability. The second research question was examined by conducting a one-way ANOVA on the gain scores the NWG and DWG achieved on the pretests and posttests. To address the third research question, the responses to the prompts which determined the layout of reflection logs were subjected to the interpretational analysis of the frequency counts of similar themes (Tesch, 1990).

### Impact of e-PA on the higher-level and lower-level skills in genre-based writing tasks

#### Feedback points on descriptive writing tasks

Descriptive statistics and the tests of normality were examined with the feedback points on six descriptive writing task outcomes.

As displayed in Table 2, the feedback points on higher-level components were much smaller on average than the feedback points on lower-level components. Moreover, they had a slower declining pattern (i.e., progress) from Task 1 to Task 6 than the feedback points on lower-level components, among which the component of word choice and grammar showed the most noticeable progress (*M*_task 1_ = 20.97 ± 1.10 to *M*_task 6_ = 9.11 ± .70) over 6 weeks.

**Table 2:** Descriptive writing tasks: descriptive statistics

Task	Statistics	Higher-level skills		Lower-level skills			Total
		Organization	Development	Sentence structure	Word choice and grammar	Mechanics	
1	Mean Std. deviation	7.10 .89	6.18 .59	17.94 1.10	20.97 1.10	13.07 .40	14.46 .87
2	Mean Std. deviation	7.04 1.94	6.64 .53	17.50 .90	18.00 .90	9.09 .42	11.65 .76
3	Mean Std. deviation	5.01 .91	4.81 .60	14.09 1.96	16.09 .88	6.70 1.54	9.34 1.03
4	Mean Std. deviation	4.90 1.50	4.67 .54	12.32 .98	15.32 .90	6.30 .44	8.70 .82
5	Mean Std. deviation	4.87 1.20	4.09 .42	8.80 1.53	10.72 .44	4.70 .52	6.63 .69
6	Mean Std. deviation	4.81 1.42	4.83 .43	8.20 1.40	9.11 .70	4.20 .90	6.23 .94

The conducted tests of normality were reported in Table 3. The ratios of skewness and kurtosis were outside the ± 2.00 interval, which retained the normality of the data (George and Mallery, 2010). The assumption of homogeneity of variances for Tasks 1 to 6 was also met, referring to the indices of Levene’s test of equality of error variances.

**Table 3:** Testing the normality assumption in feedback points on descriptive writing tasks

Task	Skewness		Kurtosis		Levene	df	Sig
	Statistic	Ratio	Statistic	Ratio	Statistic		
1	− .693	− 1.57	1.261	1.46	3.139	50	.051
2	.581	1.31	− .012	.012	1.960	50	.150
3	.482	1.09	1.101	1.285	.482	50	.201
4	.273	.612	− .379	− .443	2.613	50	.082
5	− .121	− .275	1.198	1.393	2.613	50	.063
6	− .610	− .073	.191	.229	1.813	50	.172

A set of five RM ANOVAs (corresponding to the number of higher-level and lower-level components in the WVDE rubric) was run to further explore the significance of the observed differences in the feedback points across the lower- level and higher-level skills (i.e., intragroup difference) in descriptive writing tasks completed by DWG participants over the 6 weeks (i.e., intergroup difference) (Table 4).

**Table 4:** RM ANOVA for higher-level and lower-level writing skills in descriptive writing tasks

Component	Sum of squares	df	Mean square	*F*	Sig.	Partial η^2^
*Organization*						
Between group	508.760	1	208.760	74.075	.000	.061
Within group	12.677	5	2.535	1.486	.039	.042
*Development*						
Between group	352.667	1	152.667	48.659	.020	.085
Within group	126.708	5	25.342	10.741	.000	.054
*Sentence structure*						
Between group	748.167	1	348.167	331.700	.000	.197
Within group	33.833	5	6.767	3.054	.015	.169
*Word choice and grammar*						
Between group	4056.000	1	2056.000	195.312	.000	.242
Within group	48.875	5	9.775	2.015	.036	.168
*Mechanics*						
Between group	2948.167	1	1848.167	152.037	.000	.176
Within group	91.208	5	18.242	1.893	.006	.182

In addition to the normality of the data and homogeneity of variances reported in Table 3, the RM ANOVA assumes the homogeneity of covariance matrices and the assumption of sphericity. The results of the Box’s *M* statistics (*M* = 14.197, *p* = .350 > .001) indicated that the assumption of equivalence of covariance matrices was retained. Also, Mauchly’s test of sphericity was run which suggested that the assumption of sphericity was not violated, *χ*^2^(14) = .142, *p* = .627 > .001.

The results of the RM ANOVAs were two-fold. Firstly, they indicated the significant progress of the DWG participants in all components of descriptive writing over the 6-week course of intervention. Secondly, they reported significant progress in the lower-level components of sentence structure (*F* (1, 5) = 331.700, *p* = .000, η^2^ = .197), word choice and grammar (*F* (1, 5) = 195.312, *p* = .000, η^2^ = .242), and mechanics (*F* (1, 5) = 152.037, *p* = .000, η^2^ = .176), at the cost of moderate progress of the higher-level components of organization (*F* (1, 5) = 74.075, *p* = .000, η^2^ = .061), and development (*F* (1, 5) = 48.659, *p* = .020, η^2^ = .085) (cf. calculating effect size in Lenhard and Lenhard, 2016).

#### Feedback points on narrative writing tasks

To examine the distribution of feedback points on lower-level and higher-level components of narrative writing tasks, a similar procedure was carried out. The descriptive statistical reports are summarized in Table 5.

**Table 5:** Narrative writing tasks: descriptive statistics

Task	Statistics	Higher-level skills		Lower-level skills			Total
		Organization	Development	Sentence structure	Word choice and grammar	Mechanics	
1	Mean Std. deviation	13.10 .83	15.18 .99	19.14 1.61	28.17 .70	18.01 .70	18.72 1.82
2	Mean Std. deviation	11.04 1.04	10.64 .43	18.80 .81	26.80 .94	15.29 .49	16.51 .99
3	Mean Std. deviation	9.11 .91	8.81 1.60	14.01 1.36	23.99 .68	10.70 1.04	13.32 1.73
4	Mean Std. deviation	9.40 1.00	7.67 .24	11.82 .92	20.02 .60	7.30 .34	11.24 .88
5	Mean Std. deviation	7.87 1.25	6.09 1.22	10.50 1.20	18.12 .94	4.70 .62	9.45 .94
6	Mean Std. deviation	6.81 1.02	4.13 .33	8.00 1.00	10.01 .77	2.20 .50	6.23 .94

As it can be seen in Table 5, the feedback points on the higher-level and lower-level components of narrative writing tasks showed similar proportional distribution but with overall larger quantities than those on descriptive writing tasks.

Moving forward from Task 1 to Task 6, the feedback points on higher-level components were less in number than those on lower-level components. Also, they had a task-wise descending pattern much slower than the feedback points on lower-level components. Similar to the distribution of feedback points on descriptive writing tasks, the component of word choice and grammar had the highest level of progress (*M*_task 1_ = 28.17 ± .70 to *M*_task 6_ = 10.01 ± .77).

Table 6 reports the tests of normality for the feedback points on narrative writing tasks. The normality of the data was met due to the ratios of skewness and kurtosis being outside the ± 2.00 interval. The assumption of homogeneity of variances of the data was retained for Tasks 1 to 6, referring to the indices of Levene’s test of equality of error variances.

**Table 6:** Testing the normality assumption in feedback points on narrative writing tasks

Task	Skewness		Kurtosis		Levene	df	Sig
	Statistic	Ratio	Statistic	Ratio	Statistic		
1	1.176	.479	− .108	− .098	2.119	50	.901
2	.563	1.001	.907	1.202	2.900	50	.061
3	1.009	.558	.976	1.117	1.002	50	.201
4	1.861	.303	1.821	.599	1.403	50	.826
5	.863	.653	.734	1.485	1.091	50	.910
6	.950	.593	.987	1.105	1.510	50	.072

Another set of five RM ANOVAs was run to examine the significance of the differences in the feedback points across the lower- and higher-level components of narrative writing tasks completed by NWG over the 6-week intervention in the e-PA system (Table 7). The assumption of the homogeneity of covariance matrices was retained after running the Box’s *M* statistics (*M* = 90.119, *p* = .910 > .001) and the Mauchly’s test of sphericity, *χ*^2^(14) = .880, *p* = .505 > .001, reported that the assumption of sphericity was preserved.

**Table 7:** RM ANOVA for higher-level and lower-level writing skills in narrative writing tasks

Component	Sum of squares	df	Mean square	*F*	Sig.	Partial η^2^
*Organization*						
Between group	210.333	1	112.067	21.598	.032	.068
Within group	10.042	5	10.042	26.467	.000	.072
*Development*						
Between group	552.667	1	210.667	54.659	.000	.075
Within group	26.708	5	25.342	4.741	.000	.084
*Sentence structure*						
Between group	870.167	1	302.167	169.700	.000	.190
Within group	53.833	5	26.767	3.054	.015	.169
*Word choice and grammar*						
Between group	4056.000	1	2006.000	174.075	.000	.212
Within group	48.875	5	9.775	2.015	.036	.168
*Mechanics*						
Between group	1538.167	1	748.167	114.037	.000	.106
Within group	91.208	5	10.242	9.893	.106	.182

As Table 7 indicates, the obtained results were similar to the previously conducted RM ANOVAs. Accordingly, the findings supported significant progress of NWG participants in all components of narrative writing from Task 1 to Task 6. While their progress was moderate on the higher-level components of organization (*F* (1, 5) = 21.598, *p* = .032, η^2^ = .068) and development (*F* (1, 5) = 54.659, *p* = .000, η^2^ = .075), they had a major progress in lower-level components of sentence structure (*F* (1, 5) = 169.700, *p* = .000, η^2^ = .190), word choice and grammar (*F* (1, 5) = 174.075, *p* = .000, η^2^ = .212), and mechanics (*F* (1, 5) = 114.037, *p* = .000, η^2^ = .106).

### Impact of e-PA on genre-based writing development

To address the second research question which examined the impact of e-PA on the genre-based writing improvement by NWG and DWG participants, the pretest and posttest results were co-rated by the researchers with a reference to the WVDE writing rubric with strong inter-rater reliability (Cronbach’s α = .882).

According to Table 8, the descriptive analysis of the average scores indicated the notable improvement in both groups from the pretests to posttests. However, the results of one-way analysis of covariance (ANCOVA) indicated that the observed difference between the two groups’ gain scores was insignificant (*F* (1, 55) = 61.98, *p* = .098, η^2^ = .012), interpreting the mutual benefits of NWG and DWG participants from the writing e-PA platform.

**Table 8:** Descriptive statistics: pretest and posttest scores

Group	Test	Mean	Std. deviation	95% CI
DWG	Pretest	3.19	.65	[2.29–5.09]
	Posttest	5.10	.51	[4.24–6.00]
NWG	Pretest	3.65	.55	[2.02–5.55]
	Posttest	5.85	.80	[4.54–5.96]

#### Reflection log analysis

The third research question which explored the potential impact of writing e-PA on EFL learners’ engagement at behavioral, emotional, and cognitive modes was addressed by collecting qualitative data through the in-depth content analysis of the reflection logs posted on the participants’ Moodle profiles. The participants’ behavioral mode of engagement in writing e-PA was defined in terms of the overall length of time every participant self-reported on writing their tasks, and the frequency of revisions s/he carried out after researchers’ corrective feedback. By comparison, the average of time the DWG and NWG participants spent on completing assignment task was almost identical with an insignificant difference, *M*_DWG_ = 23.18 ± 2.38 min, and *M*_NWG_ = 23.90 ± 3.18 min (Pearson *χ*^2^ (1298) = .70, *p* = .190, Cramer’s *V* = .16, interpreting a weak effect size). The frequency of revisions was also similar by number and proved to be statistically insignificant (*F*_DWG_ = 4.01 ± .36), and (*F* _NWG_ = 4.90 ± .51) (Pearson *χ*^2^ (268) = .59, *p* = .070, Cramer’s *V* = .30, interpreting a weak effect size).

The participants’ emotional mode of engagement in writing e-PA was conceptualized through a prompt question asking for the participants’ emotional experience in completing every writing task. The major extracted themes were *novelty* (*N* = 79), *low anxiety* (*N* = 65), and *fun* and *enjoyment* (*N* = 54). To the majority of the participants in both groups keeping e-portfolios and writing reflection logs were their first experience filled with enjoyment. They frequently referred to the convenient and stress-free environment of the e-PA platform that let them concentrate and plan to write or revise at their own pace.

The cognitive mode of engagement in writing e-PA was defined in terms of a prompt that required the participants to elaborate on their learning experience in every task completion. The four major extracted themes were *the applicability and usefulness of teacher feedback* (*N* = 110), *overall satisfaction with writing improvement* (*N* = 95), the *unexpected divergence between teacher feedback and students’ self-assessment* (*N* = 60), and *teacher linguistic bias or prioritization towards certain writing features* (*N* = 33). The participants in both groups frequently appreciated keeping an e-portfolio as a source of motivation to work harder and to write more. Also, they believed this encouragement caused their gradual progress in completing the writing tasks and resulted in observable improvement. Moreover, they acknowledged the usefulness and practicality of the teacher feedback in redrafting and completing future tasks.

To express their objections, the participants were critical of the differences they observed between their self-perceived strengths and weaknesses in writing and the scope of teacher feedback. For instance, a participant in DWG expressed her disappointment by writing “I am sure I know many things about conjunctions, but why I got so many notes and comments on them today!”, or another participant in NWG blamed the too detailed and large number of feedback points by writing “who can remember all these exceptions and rules? Not me!”. The participants’ negative perceptions were also directed to the prioritization in teacher feedback given to certain writing features, such as choice of words. Such ‘sensitivity’ or linguistic bias was mocked or criticized in some reflection logs. For instance, a participant in DWG blamed it by writing “It seems the focus is more on using big adjectives than correct sentences.”, or another participant in NWD showed his disappointment by stating “I wished I had a good comment on the story that I wrote. It was the funniest thing!”.

## Discussion

To sum up the findings in this study, we found out that when EFL learners actively engaged in writing e-portfolios, they could moderately improve the higher-level skills of writing, such as development and organization of genre-related ideas, while this progress could become most noticeable in lower-level skills of writing such as sentence structure, word choice and grammar, and mechanics of writing. Moreover, it was found that EFL learners’ achievement on narrative and descriptive writing tasks was equally and positively affected by the writing e-PA system. Finally, the in-depth analysis of the three modes of learner engagement in the writing e-PA triangulated the statistical findings and suggested overriding similarities between the descriptive and narrative writers in terms of the length of writing time and frequency of revisions, positive emotional feedback, overall writing improvement, and raising a critical approach to the scope and quality of teacher feedback in the e-PA platform.

The discussion of the first research question is two-fold. On the one hand, the participants’ progress in their genre-based writing can be argued from the perspective of Vygotsky’s social constructivist model (1987) underlying the alternative assessment methods such as PA. Accordingly, the L2 learners can co-construct their feedback literacy through ongoing observation, imitation, and dialogue in the course of writing PA (Price et al. 2010). Embodied in the working e-PA system, the participants in this study could systematically use their writing output as their learning input, and actively engage in the writing process. Moreover, the e-PA provided the participants with adequate time and comfort *to acquire* writing through regular writing and revising. On the other hand, the findings indicated that while the teacher feedback improved the lower-level skills of writing significantly, it affected the higher-level skills of writing to a limited degree. This finding might be argued from the cognitive psychology perspective, which underlies the cognitive operations the students undertake to analyze the feedback. Accordingly, how the recipients of the feedback *make sense* of the feedback is central to *how well* they can use the feedback in subsequent revision and improvement of writing (Sandiford and Macken-Horarik, 2020). Moreover, the meaning potential of the feedback is partially dependent on the comprehensibility of *the content* of the received feedback. The more cognitively complex the content, the lower the feedback uptake would be. Therefore, in this study, the feedback which focused on the higher-level skills in genre-based writing such as organization, coherence, or the narrative craft had a slimmer chance of comprehensibility than the feedback on the lower-level skills such as rhetorical effectiveness or grammaticality in writing.

The findings in this study are partially consistent with those in Baturay and Daloğlu (2010) who reported that keeping writing e-portfolio had no effects on improving the lower-level skills of grammar and vocabulary in writing despite the participants’ reported self-progress. In a comparative study, Baturay and Daloğlu examined 44 Turkish EFL learners divided into one group who kept writing e-portfolios and one group who were assessed through standardized tests. After triangulating the collected data from the participants and the teachers, they concluded that e-portfolio did not make any significant difference in the two groups’ learning gains. The unexpected results could be due to a rather small sample size of the groups, and a few writing tasks completed in their study. The findings in this study are also partially supported by Roohani and Taheri (2015) and Halim and Lestari (2019). Roohani and Taheri (2015) examined the potential effects of PA on improving the expository genre of academic writing. They conducted experimental research with 44 Iranian EFL university students and reported the positive role of PA in improving the higher-level skills of ‘focus’, ‘support’, and ‘organization’ in student expository writing. Yet, they found weak and temporary impacts of writing PA on students’ lower-level skills of words choice and conventions of expository writing. In a case study of the challenges, the Indonesian EFL teachers might face in running the PA, Halim and Lestari (2019) also reported the EFL teacher’s difficulties in supervising the student peer and self-assessment and considerable improvement in students’ descriptive writing despite their low rate of engagement.

The PA research literature on L2 narrative writing has dated back to the 1990s. In a case study with 22 EFL learners, Shober (1996) conducted a 12-week PA and reported that only 68 percent of the participants demonstrated improvement in their narrative writing performance. Twenty-seven percent of the students’ gain scores remained unchanged, and a single student even had 5% decrease in her final score. Shober (1996) concluded that portfolio assessment was deficient and ineffective as an evaluation tool. In another case study, Gearhart et al. (1992) adopted a portfolio-based approach to formatively assess the narrative writing improvement of 35 English-speaking elementary school students. They raised several critical issues on the efficiency of PA such as the controversial *scorability* of the portfolio and its utility as a large-scale assessment.

The discussion of the second research question to some extent overlaps with the first research question. The findings indicated the participants’ overall genre-based writing improvement in the e-PA which was echoed in their pretest-posttest summative assessment. Their successful performance is argued by a reference to the learner-centered nature of PA. PA is one of the most authentic and practical assessment methods which simulates the students’ natural practice to save a written assignment and to take a second look at it before submission. In this sense, e-PA is a flexible assessment tool in the L2 learning context, through which the L2 learners’ strengths and weaknesses are recorded through writing portfolios. In other words, e-PA provides evidence of students’ acquiring the target skills in the most observable way (Lam, 2019).

The discussion of the third research question is anchored in the critical concept of learner agency and reflective thinking as the major by-products of writing e-PA (Carless, 2011). Through the collection, selection, and reflection procedures, the L2 learners grow independence, self-assessment, and critical thinking to prepare their e-portfolios. The subsequent reflection logs on the teacher feedback entail the three phases of projection, retrospection, and revision (Yancey, 1998) which enhance the L2 learners’ metathinking and active monitoring of their works-in-progress. The findings on the third research question indicated the participants’ active behavioral engagement in spending time to prepare the interim drafts and to redraft within the e-PA framework. Such a dynamic self-regulated learning practice has been supported by several researchers. Romova and Andrew (2011), for instance, adopted a multi-draft e-PA approach to teaching and assessment of academic writing with 41 multicultural EFL learners. The authors triangulated data from the focus group reflective journals and interviews to report the participants’ growing interests in writing as a recursive process that demands self-editing and awareness of the target genre and discourse knowledge.

Regarding the emotional engagement of participants, the majority of the students agreed upon the novelty, low-stress environment, and enjoyment they experienced in writing in the e-PA system. The findings corroborate those in Afrianto (2017), Lam (2019), and Steen-Utheim and Hopfenbeck (2018) who reported the impacts of writing PA on the students’ confidence, motivation, and positive learning attitudes. Yet, several studies provided evidence that portfolios were underrated by the L2 learners as inefficient, boring, confusing, and challenging (Zhang and Hyland, 2018).

The participants’ cognitive engagement in the writing e-PA system was operationalized by their self-evaluation of the learning experience and the quality of teacher feedback. Despite insisting on the positive role of e-PA in improving their writing skills, the participants brought up the issue of teacher linguistic bias as well as the detailed and wide scope of teacher feedback. They were dissatisfied with several mismatches between teacher formative assessment and their self-assessment. The findings were in line with some qualitative research studies which reported students’ low appreciation, misinterpretation, or negative reaction to the teacher formative assessment in writing PA (Carless, 2011; Price et al. 2010).

## Conclusion and limitations

Current trends in language assessment are experiencing a paradigm shift from the standardized testing system to the alternative assessment of specific language tasks such as genre-based academic writing. The educators and language teaching practitioners in L2/EAP settings commonly search for a model of assessment that can properly highlight students’ strengths and voices rather than expose their weaknesses. The findings of this study suggest that e-PA can be one of the solutions. PA is a flexible and user-friendly tool that integrates teaching and assessment with all the benefits that an educational ICT interface such as Moodle might offer to the L2 teachers and learners. In other words, e-PA can be a platform to scaffold both L2 learner digital literacy and self-regulated language learning.

The arguments in this study are tentative due to some ecological and methodological limitations. Major restrictions were caused by the surge of the COVID-19 pandemic which affected the sampling procedure, the number of treatment sessions, and the researchers’ follow-up communications. The next related limitation was the non-random convenience sampling method which was carried out to select a group of EFL learners who were committed to attending all the virtual sessions and completing all the required tasks. Thus, the findings in this study should be used cautiously with the L2 learners of lower degrees of enthusiasm, task engagement, or digital literacy. Future researchers may incorporate the collaboration of participants in drafting and revising their written scripts or/and peer assessment in writing e-PA to expand the scope of this study. Since no analytical analysis was conducted on the student revision performance and their successful application of received feedback, it can also be a demanding topic for future research.

## Data Availability

Please contact the authors for data requests.
